# Replacement of Nitrite in Meat Products by Natural Bioactive Compounds Results in Reduced Exposure to N‐Nitroso Compounds: The PHYTOME Project

**DOI:** 10.1002/mnfr.202001214

**Published:** 2021-08-27

**Authors:** Simone G. van Breda, Karen Mathijs, Harm‐Jan Pieters, Virág Sági‐Kiss, Gunter G. Kuhnle, Panagiotis Georgiadis, Giovanna Saccani, Giovanni Parolari, Roberta Virgili, Rashmi Sinha, Gert Hemke, Yung Hung, Wim Verbeke, Ad A. Masclee, Carla B. Vleugels‐Simon, Adriaan A. van Bodegraven, Theo M. de Kok, the PHYTOME consortium

**Affiliations:** ^1^ Department of Toxicogenomics GROW‐school for Oncology and Developmental Biology Maastricht University Medical Center P.O. Box 616, 6200 MD Maastricht the Netherlands; ^2^ Department of Food & Nutritional Sciences University of Reading Reading UK; ^3^ National Hellenic Research Foundation Institute of Biology Medicinal Chemistry and Biotechnology Athens Greece; ^4^ SSICA‐Experimental Station for the Food Preserving Industry Parma Italy; ^5^ Division of Cancer Epidemiology & Genetics National Cancer Institute National Institutes of Health Bethesda MD USA; ^6^ Hemke Nutriconsult Prins Clauslaan 70, 5684 GB Best The Netherlands; ^7^ Department of Agricultural Economics Ghent University Coupure links 653 Gent 9000 Belgium; ^8^ Division of Gastroenterology‐Hepatology Department of Internal Medicine Maastricht University Medical Center Maastricht The Netherlands; ^9^ Zuyderland Medical Center Sittard‐Geleen The Netherlands

**Keywords:** colorectal cancer risk, gene expression, genotoxicity, human dietary intervention study; N‐nitroso compounds

## Abstract

**Scope:**

It has been proposed that endogenously form N‐nitroso compounds (NOCs) are partly responsible for the link between red meat consumption and colorectal cancer (CRC) risk. As nitrite has been indicated as critical factor in the formation of NOCs, the impact of replacing the additive sodium nitrite (E250) by botanical extracts in the PHYTOME project is evaluated.

**Method and Results:**

A human dietary intervention study is conducted in which healthy subjects consume 300 g of meat for 2 weeks, in subsequent order: conventional processed red meat, white meat, and processed red meat with standard or reduced levels of nitrite and added phytochemicals. Consumption of red meat products enriched with phytochemicals leads to a significant reduction in the faecal excretion of NOCs, as compared to traditionally processed red meat products. Gene expression changes identify cell proliferation as main affects molecular mechanism. High nitrate levels in drinking water in combination with processed red meat intake further stimulates NOC formation, an effect that could be mitigated by replacement of E250 by natural plant extracts.

**Conclusion:**

These findings suggest that addition of natural extracts to conventionally processed red meat products may help to reduce CRC risk, which is mechanistically support by gene expression analyses.

## Graphical abstract

The PHYTOME project develops innovative meat products in which the food additive nitrite was replaced by natural compounds originating from fruits and vegetables. These biologically active compounds (phytochemicals) contribute to improved gut health. Subjects consuming red processed meat enriched with phytochemicals formed lower levels of potentially carcinogenic N‐nitroso compounds in their gut, which may reduce their risk on colorectal cancer.

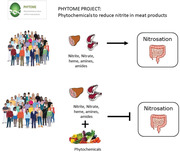

## 1 Introduction

The safety of processed meat consumption is being debated already for many years. According to several reports of the World Cancer Research Fund,^[^ ^1^, ^2^ ^]^ there is convincing evidence that the consumption of red and particularly processed meat is associated with cancer risk. Additionally, the International Agency on Research on Cancer has classified processed meat as carcinogenic to humans (Group 1 carcinogen), based on sufficient evidence in humans that the consumption of processed meat causes colorectal cancer (CRC).^[^ ^3^ ^]^ Others argue however, that meat and meat products form a conventional part of the human diet and also contribute to the health of consumers in view of the supply of essential amino acids, iron, zinc, selenium, vitamin B6 and B12, and vitamin D. According to Hodgson et al.,^[^ ^4^ ^]^ the consumption of red meat also results in a reduced blood pressure and may thus contribute to reduced risk of cardiovascular diseases. As a consequence, there has been quite some debate on how to reach consensus on the healthiness of red and processed meat.^[^ ^5^ ^]^

In order to find a mechanistic explanation for the link between meat consumption and CRC risk, the formation of food preparation‐related compounds, such as heterocyclic amines (HCAs) and polycyclic aromatic hydrocarbons (PAHs), have been investigated. Also the exposure to endogenously formed N‐nitroso compounds (NOCs) has been suggested as a potentially relevant risk factor since red meat is known to stimulate NOC formation in the colon.^[^ ^6^, ^7^, ^8^, ^9^ ^]^ Meat in general is a source of NOC precursors in the form of amines and amides, and nitrate and nitrite in particularly salt‐preserved meat.^[^ ^10^ ^]^ Furthermore, haem protein present in red meat is found to catalyse endogenous nitrosation.^[^ ^9^, ^11^ ^]^ In addition, meat products, and especially processed meat products, already contain pre‐formed NOCs.^[^ ^12^ ^]^ As most NOCs have mutagenic and genotoxic properties, explaining their carcinogenic effect in test animals,^[^ ^13^ ^]^ they may also contribute to CRC development in humans. However, evidence from epidemiological studies is inconsistent and methodological issues are apparent. In particular, quantifying individual exposure to NOCs is difficult. In addition, the presence of other dietary variables that can affect the formation of NOCs, such as intake of amines and amides, vegetables and fibre, are not taken into account. Also, studies often do not control for confounding factors such as haem iron and saturated fat.^[^ ^14^, ^15^ ^]^ Furthermore, genetic variation in the study population could result in differences in responses to meat consumptions and long‐term CRC risk, which could explain the observed inconsistencies.^[^ ^15^ ^]^ Moreover, gene expression changes associated with NOC exposure could play a part in the carcinogenic process. Indeed, human dietary intervention studies have shown that red meat intake‐induced faecal water genotoxicity correlate with pro‐carcinogenic gene expression changes in human colonic tissue.^[^ ^16^, ^17^, ^18^, ^19^ ^]^

Nitrite is added to meat to control the growth of pathogenic bacteria, to prevent rancidity, and to create the characteristic pink color of cured meats that is appreciated by consumers.^[^ ^20^, ^21^, ^22^ ^]^ In view of its role in the formation of NOCs, the use of nitrite in meat products is limited by the European Commission to a maximum of 150 ppm.^[^ ^21^ ^]^ In order to find alternative meat processing techniques that limit the use of nitrite while guaranteeing microbiological safety, the PHYTOME project (www.phytome.eu) explores the use of biologically active compounds from botanical sources, to replace or reduce nitrite in meat processing.^[^ ^23^ ^]^ These biologically active compounds, also referred to as phytochemicals, may have antimicrobial activity, and include a wide range of chemical classes such as tocopherols, flavonoids, carotenoids, glycol alkaloids, and vitamins, all from natural sources. These compounds may exert their beneficial action via different mechanisms, including the inhibition of the formation of NOC, effects at the level of kinetics of carcinogenic compounds in the colon, and at the level of cellular protection.^[^ ^24^, ^25^, ^26^ ^]^ Due to synergistic interactions, specific combinations may be more effective than single compounds.^[^ ^27^, ^28^ ^]^ Some of these compounds also possess antimicrobial activity and may therefore contribute to microbiological safety of the product.^[^ ^29^ ^]^ Natural compounds are also known to protect the gut from the induction of for instance oxidative genetic damage by other dietary factors.^[^ ^27^, ^28^, ^30^ ^]^ In order to replace or reduce nitrite in processed meat without losing their properties, many scientific studies have been carried out the last 20 years, and several have shown that the use of biologically active compounds is promising. It was demonstrated that essential oils containing several bioactive compounds such as eugenol and cinnamaldehyde possess antimicrobial activity while added to fresh meat and meat products.^[^ ^31^ ^]^ In addition, specific antioxidants such as grape seed extract and propyl gallate, were able to reduce oxidation in cooked, frozen, reheated ground beef patties.^[^ ^32^ ^]^ Furthermore, a number of food‐derived antioxidative phenolic compounds among which theaflavin 3,39‐digallate, epicatechin gallate, rosmarinic acid, and naringenin reduced formation of HCAs in beef patties.^[^ ^33^ ^]^

Therefore, the aim of the present study is to evaluate the effect of consumption of processed red meat products with reduced levels of nitrite and enriched with phytochemicals on the exposure to NOC and on several biomarkers of effect in a human dietary intervention study. We hypothesize that consumption of processed red meat products with reduced‐nitrite levels and added phytochemicals decreases the exposure to NOC and the associated induction of DNA damage in the large intestine of healthy volunteers. Before and after the dietary intervention, gene expression changes may reflect the molecular responses that are involved in cancer preventive action of the added phytochemicals or reduced exposure to NOC. As it is known that nitrate in drinking water may be converted by oral bacteria into nitrite and thus contribute to the formation of NOCs in the large intestine, particularly when high drinking water nitrate is combined with high intake of red and processed meat,^[^ ^34^, ^35^, ^36^ ^]^ this study also aims to quantify the effect of drinking water nitrate on NOC formation in healthy subjects consuming meat products with and without replacement of nitrite.

## 2 Experimental Section

### 2.1 Study Population

Participants were recruited using advertisements in local newspapers, by social media, and by means of flyers posted at public locations. Volunteers met with the principal investigator at Maastricht University, were provided an information brochure, and were given 1 week to decide whether to participate in the study. Healthy subjects of both sexes were selected based on predefined inclusion criteria and randomly and blindly assigned to one of the different experimental groups. The randomization procedure was as follows: subjects received a subject number given in sequence based on the date of signing the informed consent. Next, subjects were divided based on study start rotating between group 1 and 2. The participant flowchart is visualized in Figure S1, Supporting Information. Both healthy men and women with a body mass index between 18 and 25, in the age of 18 to 70 years were included in the study. Exclusion criteria included alcohol abuse up to 6 months before participation in this research, presence or symptoms of any diseases related to the gastrointestinal tract, kidney, liver, heart, lungs; the endocrine or metabolic system; presence of anemia, HIV infection or hepatitis; use of antibiotics and other medication over the last 3 months; smoking, adhering to a vegetarian or vegan diet, pregnancy, and participating in other intervention studies during this intervention period. The protocol of the study was in accordance with the guiding principles of the Declaration of Helsinki, approved by the local Medical Ethics Review Committee of the Maastricht University Medical Centre+ (registration number: NL43956.068.13), and registered at Clinical trials.gov under identifier: NCT04138654. All subjects gave written informed consent.

### 2.2 PHYTOME Meat Products

PHYTOME meat products, including cooked sausages, raw and cooked ham, dry fermented sausages, and dry cured ham, have been prepared for the human dietary intervention study at two levels of nitrite: standard‐nitrite (group 1) and reduced‐nitrite (group 2) levels. In group 1, preservatives were added according to standard manufacturing practices and European rules,^[^ ^37^ ^]^ while in group 2, nitrite reduction or elimination was obtained in safety conditions and preserving the traditional sensory traits of the end product. Both groups were enriched with selected combinations of natural antioxidants and bioactive molecules delivered by plant extracts. These were carefully selected based on a number of criteria, that is, the level of scientific evidence for their antioxidant and/or chemopreventive, and antimicrobial properties; some basic requirements for these candidate extracts such as natural origin of all extracts, their commercial availability, and compatibility with technological requisites. Next, extracts were tested in several combinations, according to good manufacturing processes, in different trial versions of the innovative meat products. Furthermore, the extracts should not negatively affect quality and/or sensory traits of the meat. Commercial extracts from *Poligonum cuspidatum*, *Sophora japonica*, green tea, white grape, rosemary, oregano, sage, *Melissa*, and acerola were added in meat mince or curing brines, as natural sources of polyphenols and ascorbic acid. Depending on the meat item, manufacturing techniques were adapted to add the natural extracts to processed meats in order to provide a polyphenol intake per serving that is reported to reduce cancer risk.^[^ ^38^, ^39^ ^]^ At the same time, obtained PHYTOME meat products showed no or limited impact on overall sensory acceptability.^[^ ^40^ ^]^ An overview of the formulation of PHYTOME meat products is provided in **Table** **1**. As reported in Saccani et al.,^[^ ^40^ ^]^ final meat products show different concentrations of polyphenols and ascorbic acid, due to a combined effect of the different processing techniques, the added extracts, and the level of added nitrite. Meat processing techniques were modified to optimize the final concentrations of bioactive compounds and to guarantee the quality and sensory traits of the meat product. In dry and cooked sausages concentrations around 2–2.5 (as g kg^−1^ gallic acid equivalents), and 0.5 g kg^−1^, respectively, were detected. In the case of dry cured hams, salted by means of brine vacuum impregnation (BVI)^[^ ^41^ ^]^ polyphenols and ascorbic acid concentrations around 1–1.5 and 0.4 g kg^−1^, respectively were obtained. Lower amounts of polyphenols and ascorbic acid were found in cooked and raw hams processed by brine injection (<0.5 and <0.1 g kg^−1^, respectively). Processing techniques have been furtherly revised to ensure safety of meats processed without nitrite or with added nitrite <50 mg kg^−1^.^[^ ^42^ ^]^ In the case of dry sausage (25 mg kg^−1^ added nitrite), dry sausage southern style and dry cured ham (both without added nitrite), an early cold drying treatment (0–3 °C) has been applied, to allow a_w_ reduction and pH decrease (in dry sausages) in safety conditions.^[^ ^43^, ^44^ ^]^

**Table 1:** PHYTOME meat product formulations: level of nitrite and nitrate (mg kg^−1^) and natural extracts (g kg^−1^) added during meat manufacturing

Meat	Added nitrite/nitrate [mg kg^−1^]		Natural extracts [g kg^−1^]^a)^						
	Standard‐nitrite PHYTOME meat	Reduced‐nitrite PHYTOME meat	Standard‐nitrite and reduced‐nitrite PHYTOME meat						
			Polygonum	Rutin/Sophora	Green tea	Origanox	White grape	Rose‐mary	Acerola
Cooked ham	100/0	25/0	0.1	0	0	0	0	0	2.5
Raw ham	150/150	75/0	0.3	0	1.5	0.75	0.75	0.3	2.2
Cooked sausage	150/0	25/0	0.1	0.5	1.2	0.65	0.65	0.65	2.5
Dry sausage	150/150	25/0	0.05	0.25	0.65	0.65	0.65	0.65	2.5
Dry cured ham	150/150	0/0	0.08	0.4	1.25	1.25	1.25	1.25	1.25
Dry sausage Southern style	80/150	0/0	0.05	0.25	0.65	0.65	0.65	0.65	2.5

^a)^ Botanic source or trade name, main bioactive molecules composition %, supplier:

*Polygonum Cuspidatum* root, Resveratrol 98%, Nutraceutica, Italy;

*Sophora Japonica*, Rutin 98%, Indena, Italy;

Green tea, Epigallocathechingallate (EGCG) 40%, Indena, Italy;

Origanox WS‐T, Polyphenols 30% as gallic acid from oregano, sage, *Melissa*, Frutarom, Italy;

White grape NutriPhy, Polyphenols 95% as gallic acid, Chr Hansen, Italy;

Rosemary – Aquarox Polyphenols 15% as gallic acid,Vitiva, Slovenia;

Acerola, ascorbic acid 17%, Raps, Germany.

### 2.3 Study Design

**Figure 1:**
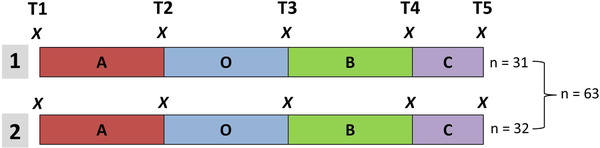
PHYTOME study design. A = processed red meat products, O = control period with only white meat; B: processed red meat products with added natural extracts; C = drinking water nitrate at acceptable daily intake (3.7 mg kg^−1^ body weight) combined with one of the three meat types; X = sampling moment at time point 1 to 5 (T1–T5). Group 1: standard‐nitrite in period B1 (standard‐nitrite PHYTOME meat); group 2: reduced‐nitrite in period B2 (reduced‐nitrite PHYTOME meat). Period A, B, and O lasted 14 days and period C lasted 7 days.

The human dietary intervention study has a parallel design as shown in **Figure** **1**. There are two study groups (1 and 2), and in each study group there are two meat intervention periods (A and B), separated by a wash‐out period (O). During intervention period “A,” participants were asked to consume the provided processed red meat products (processed red meat group), while in period “B” processed red meat products enriched with natural extracts were provided. For study group 1, the meat products of both period ”A” and “B” had standard‐nitrite levels (standard‐nitrite PHYTOME meat), while in study group 2 the nitrite levels in period “B” were reduced (reduced‐nitrite PHYTOME meat). During period “C” drinking water nitrate was increased to the Acceptable Daily Intake level (ADI: 3.7 mg kg^−1^ bodyweight). Therefore, participants received bottled drinking water each day, which was individually adjusted to the nitrate ADI level by adding appropriate amount of KNO_3_ and adjusting for the regular daily nitrate intake from other dietary sources. Throughout intervention periods “A,” “O,” and “B,” all participants received bottled drinking water with low nitrate levels (<2 mg L^−1^). Results from previous intervention studies on NOC formation indicate that a period of 15 days for each part of the study would be sufficient to detect changes in the markers to be measured.^[^ ^45^ ^]^ Gene expression changes, however, are expected to be observed earlier.^[^ ^19^ ^]^ Due to the rapid mitotic activity of the regenerative cells, the epithelial lining of the crypts and the mucosal surface of the colon is replaced every 6 to 7 days. Therefore, the wash‐out period of 14 days was considered to be sufficient to remove or reduce possible effects of the red meat intake to a base level.

An individual food package for each volunteer was compiled in consultation with a dietitian. These food packages were composed to contain all daily nutritional requirements. The results of the national food consumption survey in the Netherlands shows an average daily meat consumption of 150 g per day on average. In order to maximize intake, it was decided for intake of maximal 300 g of meat per day for a person with a body weight of 80 kg (3.75 g kg^−1^ body weight, with a maximum of 300 g per day), based on previous studies.^[^ ^19^, ^45^ ^]^ This amount is within the range of daily meat consumption in the Netherlands, and halfway between median daily intake and the 95th percentile according to the RIVM food consumption survey from 2012 to 2014 for the age group of 21–50 years.^[^ ^46^ ^]^ During the intervention period, intake of fruits and vegetables were kept at a low, but acceptable level of 50 g of vegetables and one piece of fruit per day. The food package for the processed red meat group (“A” period) contained a variety of cooked and dry‐cured red meat products (cooked ham, raw ham, cooked sausage, dry sausage, and dry cured ham), which are conventional processed meats, containing standard levels of nitrite. The food package for the PHYTOME meat (“B” period) also contained a variety of cooked and dry‐cured conventional red meat products, containing standard levels of nitrite (group 1; standard‐nitrite PHYTOME meat) or reduced levels of nitrite (group 2; reduced‐nitrite PHYTOME meat), but with the enrichment of natural extracts. This enriched meat was tested to meet all quality standards for conventional meat products. An overview of the composition of the PHYTOME meat products is provided in Table 1. The food package for the “O” period (white meat group) contained unprocessed chicken and turkey. Fish was excluded during the entire experimental period in view of the presence of high amounts of amines which could interfere with the analyses.^[^ ^11^ ^]^

At the beginning of the study and after each intervention period of 2 weeks, colonic biopsies were collected during an endoscopic examination. First, a rectal swab was taken and subsequently six small colonic biopsies were collected, transferred to an Eppendorf container and immediately stored in liquid nitrogen. Urine was collected during 24 h prior to the endoscopic examination, in a 2 L bottle. Total sample was weighted, mixed and 2 × 10 mL was stored at −80 °C until use. One faecal sample was collected 24 h prior to endoscopic examination. Total amount was weighted and a small volume was scooped out and stored at −20 °C until further analysis. About 2–5 mL of saliva sample was collected in the morning before the endoscopic examination, and stored at –20 °C until use. Blood samples were collected before endoscopy using EDTA tubes by means of a venipuncture. One tube was split over six Eppendorf containers with RNA*later* (Qiagen, Venlo, the Netherland) (0.5 mL blood with 1.3 mL RNA*later*) and stored at –80 °C until use. Faecal water, blood, and colonic biopsies were analyzed for different markers of exposure and effect. Saliva and faeces, and urine were used for analyses of the microbiome and nitrate levels, respectively, the results of which are described in a separate paper.^[^ ^47^ ^]^ Furthermore, all participants were asked to keep a food diary during the whole 7 weeks of intervention to keep track of everything they consumed (amounts per food product per meal). Participants were instructed to record their daily dietary intake during the study using an online standardized food diary from ``Voedingscentrum (https://mijn.voedingscentrum.nl) using the software program “Eetmeter” designed by the Netherlands Nutrition Centre. For each food item, the amount consumed (standard portions: number of units, glasses, cups) was recorded per day. Food diaries were processed to calculate the average daily amounts of energy and nutrients using the “Eetmeter” database.

Nitrate in drinking water may be more important in NOC formation than nitrate intake through dietary consumption.^[^ ^34^, ^35^, ^36^ ^]^ Therefore, we quantified the effect of drinking water nitrate on the formation of NOC in healthy volunteers on a high meat diet. During the first 6 weeks, participants were asked to consume bottled drinking water, which contained low nitrate levels (< 2 mg L^−1^). To establish the impact of nitrate in drinking water on the endogenous nitrosation, we included an extra intervention period of 7 days in which participants were asked to consume drinking water with high nitrate levels (“C” period) according to the ADI level (ADI: 3.7 mg kg^−1^ bodyweight) in combination with 300 g (again adjusted for bodyweight) processed red meat, white meat, or red meat with reduced‐nitrite and added natural extracts, randomly divided over all participants. They received bottled drinking water that was individually adjusted by addition of KNO_3_. Also after this intervention period, participants were asked again to collect a saliva sample, 24 h urine, and a faeces sample. The guidelines for nitrate and nitrite levels in drinking water and meat products were taken into account.

### 2.4 Faecal Water Apparent Total N‐nitroso Compound (ATNC) Determination

Faecal water was prepared after manual homogenization of the faecal material for 2 min. by ultracentrifugation at 50,000g ×*g*  for 2 h at 10 °C. The faecal water supernatant was aliquoted and stored at –20 °C until use.^[^ ^48^ ^]^ The faecal level of NOC is frequently measured as Apparent Total Nitroso Compounds (ATNC) and used as indicator of colonic endogenous nitrosation.^[^ ^9^ ^]^ ATNC concentrations were determined using chemical denitrosation with chemiluminescence detection and quantified using a NaNO_2_ standard curve, on a Ecomedics CLD‐88 with custom‐ made glassware, as described van Breda et al.^[^ ^36^ ^]^ In short, faecal water samples were thawed on ice in the dark to prevent UV‐induced denitrosation. 50 µL of faecal water was treated briefly (1 min) with preservation solution (0.1 M N‐ethylmaleimide and 0.01 M DTPA) then incubated with 50 g L^−1^ sulfamic acid for 4 min. The sample was directly injected to the purge vessel (60 °C) containing 10–15 mL reduction solution (11.11 g L^−1^ potassium iodide and 5.55 g L^−1^ iodine in 40 mL water and 140 mL glacial acetic acid). Results are expressed as µmol ATNC per L faecal water.

NOCs were measured as apparent total N‐nitroso compounds (ATNC). ATNC concentrations were determined using a chemiluminescence detector (CLD).^[^ ^28^ ^]^ Thawed faecal water samples were kept in the dark on ice and analyzed as soon as possible and within 2 h. 100 µL of faecal water sample was treated briefly with preservation solution (0.1 M N‐ethylmaleimide and 0.01 M DTPA) and then incubated with 50 g L^−1^ sulfamic acid for 1–5 min. Nitrite content forms a diazo complex with the sulfamic acid that is stable in tri‐iodide, this step is necessary to differentiate the nitrite content from the ATNC content. The sample was directly injected to the purge vessel (60  °C) containing 10–15 mL reduction solution (11.11 g L^−1^ potassium iodide and 5.55  g L^−1^ iodine in 40  mL water and 140  mL glacial acetic acid). Preservation solution was added to preserve the nitrosation state of thiols by alkylating free thiol groups and scavenging metal ions, which can cause a release of NO from nitroso‐thiols. Tri‐iodide reduction solution releases NO from nitrite, nitrosothiols, nitrosamines, iron‐nitrosylhemoglobin, and nitrosohemoglobin. ATNC contribution to the total CLD signal was determined by subtracting the nitrite response from the total response. All samples and standards were measured in duplicates.

### 2.5 Analysis of Faecal Water Genotoxicity

The human colon adenocarcinoma cell line Caco‐2 was used to test fecal water genotoxicity in the comet assay.^[^ ^19^ ^]^ Caco‐2 cells (passages 33–38) were maintained in DMEM (Sigma‐Aldrich, D5796), supplemented with 1% penicillin/streptomycin (Gibco, 15070‐063), 1% MEM NEA (Gibco, 11140‐035), 1% sodium pyruvate (Gibco, 11360‐039), and 10% FBS. Cells were harvested using 0.05% Trypsin‐EDTA (Gibco, 25300‐054). Cell cultures were incubated at 37 °C in a humidified incubator containing 5% CO_2_. For exposure to faecal water, cells were harvested by trypsinization and resuspended in growth medium containing 10% faecal water to a final concentration of 2 × 10^6^ cells mL^−1^, mixed with 50 µL faecal water, and incubated at 37 °C for 30 min. The comet assay was subsequently performed in triplicate as described by Singh et al.^[^ ^49^ ^]^ and Pflaum et al.^[^ ^50^ ^]^ with minor modifications. Comets were visualized using a Zeiss Axioskop fluorescence microscope (at 200x magnification). Randomly, 50 cells were analyzed using the Comet assay III software (Perceptive Instruments, Haverhill, UK). DNA damage was expressed as tail moment (TM, the product of tail DNA content and mean tail migration distance). From each sample an A and B slide were made which were electrophoresed on the same day in a different run. Also included during each electrophoresis run were a positive (i.e., Caco‐2 cells treated with 200 µM H_2_O_2_) and negative (i.e., Caco‐2 cells treated with DMEM) control sample in order to compensate for any inter‐electrophoresis variation.

### 2.6 DNA Adducts Analyses in Colonic Biopsies

#### 2.6.1 DNA Isolation

Colonic biopsies were disrupted using a tissue homogenizer, subsequently lysed in 500 µL digestion buffer (containing 0.5 M EDTA; 1 M Tris–HCl, pH 8.0; 10% SDS) and incubated for 1 h at 55 °C. Next, 25 µL of proteinase K (20 mg mL^−1^) (Ambion) was added. After incubation during 1 h at 55 °C, the proteinase K was inactivated for 10 min at 80 °C. RNAse A (2 µL; 100 mg mL^−1^) (Qiagen) and collagenase (25 µL; 1%) (Sigma) treatment was performed for 1 h at 37 °C. Thereupon, 500 µL of phenol‐chloroform‐isoamylalcohol (PCI; 25:24:1) (Sigma) was added. The mixture was shaken manually for 5 min, and centrifuged for 5 min at maximum speed. The upper phase was transferred to a new Eppendorf tube and the PCI step was repeated. The upper phase was collected and precipitated using 50 µL 3 M NaAc pH 5.6 and 1250 µL cold 100% ethanol for 30 min at –80 °C. After centrifugation for 30 min at maximum speed, the DNA pellet was washed using cold 70% ETOH, dried in a speed vac and dissolved in 100 µL nuclease free water. The total amount was at least 30 µg DNA, the 260/280 ratio ranged between 1.7 and 1.9, and the 260/230 ratio between 2.2 and 2.3.

#### 2.6.2 Analysis of O^6^‐methylguanine Adducts

Exposure to certain NOCs causes O^6^‐methylguanine (O^6^‐MeG) adducts formation. O^6^‐MeG DNA adducts are also mutagenic and have frequently been shown to occur in human CRC tissue.^[^ ^51^, ^52^ ^]^ Samples were analyzed using an ELISA method described by Georgiadis et al.^[^ ^53^ ^]^ In short, restriction enzymes were used to digest DNA to fragments of size expected to contain no more than one O^6^‐meG residue. Anti‐adduct antisera were used to transfer O^6^‐meG–containing fragments to a solid surface, where they were detected using anti‐ssDNA antisera. A chemiluminescence signal was finally generated by the addition of CDP‐Star substrate containing Emerald II enhancer. Chemiluminescence was measured using a Safire II Microplate Luminometer (TECAN) at 542 nm. For the quantitation of O6‐meG in unknown DNA samples, a standard curve consisting of DNA standards containing different levels of O^6^‐meG were prepared with DNA from HeLa cells. The assay has a limit of detection of 1.5 adducts per 10^9^ nucleotides using 10 µg of DNA.

### 2.7 Transcriptomics Analyses of Colonic Tissue

#### 2.7.1 Total RNA Isolation

Colon biopsies were dissolved in QIAzol (Qiagen, Venlo, The Netherlands) using a tissue disruptor. RNA was isolated according to the manufacturer's protocol l and followed by DNase I (Qiagen) treatment. Upon purification, RNA concentrations were measured by means of a NanoDrop ND‐1000 spectrophotometer (Thermo Scientific) at 260 and 280 nm. RNA quality and integrity were assessed by using automated gel electrophoresis on an Agilent 2100 Bioanalyzer system (Agilent Technologies) (average RNA integrity number: 7.1 ± 1.0). For one subject, RNA isolated at time point one was of insufficient quality and therefore not included in subsequent analyses.

#### 2.7.2 Gene Expression Microarrays

Microarray hybridization was performed on Agilent 8 × 44K whole human genome microarrays as described previously with some modifications.^[^ ^17^, ^18^ ^]^ Quality control check was done in R (www.r‐project.org) using Bioconductor, including local background correction, flagging of bad spots, controls and spots with too low intensity, log_2_ transformation and normalization. Data were normalized per batch (date of microarray hybridization) using quantile normalization. Two arrays were excluded due to bad quality. In total, data of 247 arrays were included in the analyses. Genes with less than 30% flagged bad spots were used as a pre‐processed input file. Replicates were merged using the median and missing values were imputed by k‐nearest neighbours (KNN) imputation (*N* = 15). In order to check for multicollinearity, a Variance Inflation Factor test for gender, age, BMI, phytochemicals, subject, levels of ATCN, grams of meat and batch effect using the R package HH (http://cran.r‐project.org/web/packages/HH/index.html) was carried out. Variables that do not present multicollinearity can be analyzed independently. The values were all below 4, indicating absence of multicollinearity between the variables of study. Next, hierarchical clustering on all probes (16,734) was carried out to identify potential outliers in each group. From 59 individuals, four were removed from the processed red meat group, and two were removed from the white meat group and PHYTOME meat group (Figure S1, Supporting Information).

The microarray data have been deposited in NCBI's Gene Expression Omnibus^[^ ^54^ ^]^ and are accessible through GEO series accession number GSE 147996 https://www.ncbi.nlm.nih.gov/geo/query/acc.cgi?acc = GSE147996).

#### 2.7.3 Selection of Differentially Expressed Genes Using LIMMA Analysis

A list of differentially expressed genes (DEGs) for each group compared to the other groups was generated using the Linear Model for Microarray Analysis (LIMMA) analysis from Bioconductor (https://bioconductor.org/biocLite.R) (absolute FC > 1.2; FDR corrected *p*‐values < 0.05 [FWER‐corrected using the False Discovery Rate]), correcting for sex, age, BMI, and batch of microarray hybridization between the different groups.^[^ ^55^ ^]^ LIMMA makes use of linear models to assess differential expression in the context of multifactor designed experiments. It provides the ability to analyze comparisons between many RNA targets simultaneously.

#### 2.7.4 Selection of Genes Associated with Exposure Using Linear Mixed Model Analysis

A linear mixed model analysis was carried out in order to generate lists of significant genes associated with different conditions using the lme4 R package (https://cran.r‐project.org/web/packages/lme4/index.html). This approach has been successfully used in previous studies.^[^ ^56^, ^57^ ^]^ In short, for each subject i, we performed the mixed model Yi∼α+β1Xi+β2FEi+uAi+∈i, where Xi is the variable of interest, Yi are the predictors (gene expression), FEi are the fixed effects (confounders), Ai represents the value of the random effect, α is the intercept, β1/β2 are the regression coefficients for each variable and ∈i is the residual or difference between observed values and calculated ones by the model. We applied three different linear mixed model analyses. The first one studies the effect of ATCN levels on gene expression correcting for the confounders age, gender, BMI and yes/no phytochemicals and the random effects subject, batch of RNA isolation, samples labeling, and microarray hybridization. The second model was applied to study the effect of introducing phytochemicals on gene expression. The model remains the same as the first one but correcting for ATCN levels as confounder instead of yes/no phytochemicals. The last model was implemented to analyze the effect of the interaction ATCN and yes/no phytochemicals, keeping the same confounders as before and correcting for the individual variables of ATCN and yes/no phytochemicals.

#### 2.7.5 Data Analyses: Pathway Analyses and Network Visualization of Significant Genes

Gene lists were uploaded onto the web‐tool ConsensuspathDB^[^ ^58^ ^]^ in order to identify the biological pathways that were over‐represented (FDR corrected *p*‐value < 0.01, >2 genes per pathway). Next, genes were uploaded onto the induced network modules application, in order to identify connections between the genes. Networks consisting of less than five genes were excluded. Generated networks were exported and visualized using Cytoscape.^[^ ^59^ ^]^ Fold changes of DEGs in case of LIMMA analyses or regression coefficients in case of LMM were imported and visualized on the network nodes. A selection of genes which showed a high number of interactions with other genes were explored in more detail, and their role in development of CRC was investigated.

### 2.8 Statistical Analyses

Statistical analyses were performed using IBM Statistics SPSS, version 24 (IBM, Amsterdam, the Netherlands). Normality of the data was checked by means of histograms and Q‐Q plots. Significant differences in age, BMI, meat intake, and physical activity between group 1 and 2 were analyzed using Student's *t*‐test. Homogeneity of variance was tested by Levene's test. All tests were two‐sided and a *p*‐value <0.05 was considered statistically significant. Significant differences between the different time points for intake of energy and dietary macronutrients, ATNC, O^6^‐methylguanine adducts, mean and median TM, were assessed by means of repeated measures ANOVA. In case of a significant finding (*p*‐value <0.05), between group analyses were performed by means of pairwise *t*‐tests. All tests were two‐sided and a *p*‐value <0.05 was considered statistically significant. Data are presented as the mean (SD), unless otherwise stated.

## 3 Results

### 3.1 Study Population

In total, 78 participants were recruited and included in the study. As a consequence of 15 drop‐outs, mostly due to illnesses preceeding the study start, 63 participants started the full study. Characteristics of the study population are presented in **Table** **2**. Male and female participants were equally distributed over group 1 and 2, with respectively standard or reduced‐nitrite levels in the processed red meat products and enriched for phytochemicals. Age, BMI, meat consumption, and physical activity were not significantly different between the two groups. Furthermore, no significant differences in intake of energy during the different intervention periods were observed (Table S1, Supporting Information). However, daily intake of total fats and saturated fats were significantly lower (*p* < 0.001) and intake of carbohydrates and proteins were significantly higher (*p* < 0.05, and *p* < 0.001, respectively) during the control period in which white meat was consumed, as compared to the intake of these macronutrients during the other intervention periods. This can be attributed to the difference in consumed meat type, as white meat contains relatively more proteins and less total and saturated fats.

**Table 2:** Main study population characteristics, frequencies (*n*), and means (SD)

	Total^a)^	Group 1 (standard‐nitrite period B)	Group 2 (reduced‐nitrite period B)
Participants [*n*]	63	31	32
Females [*n*]	32	16	16
Males [*n*]	31	15	16
Age [years]	25.4 (8.5)	25.9 (9.3)	24.6 (7.6)
BMI [kg m^−2^]	22.3 (2.1)	22.0 (2.1)	22.6 (2.1)
Meat intake [g per day]	254 (38)	248 (38)	259 (37)
Physical activity [h per week]	6.5 (3.8)	7.1 (4.2)	5.9 (3.3)

^a)^ No statistically significant differences between group 1 and 2.

### 3.2 Apparent Total N‐nitroso Compound Exposure

**Figure 2:**
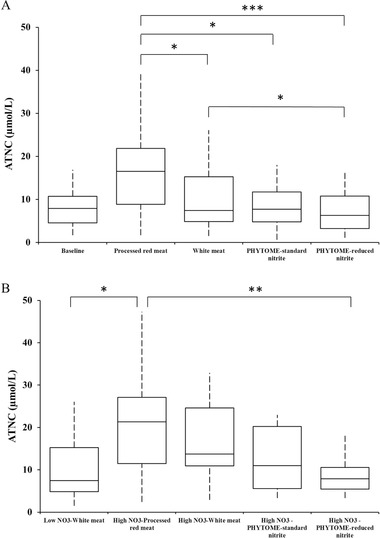
Apparent total N‐nitroso compound (ATNC) levels in faecal water (µmol L^−1^) (median, interquartile range, and range—excluding outliers) in human subjects after an intervention with daily consumption of 300 g (adjusted for bodyweight with a maximum of 300 g for a person of 80 kg) of different meat products for 2 weeks (white meat, processed red meat, processed red meat with a standard level of nitrite and added phytochemicals [standard‐nitrite PHYTOME meat], or processed red meat with a reduced level of nitrite and added phytochemicals [reduced‐nitrite PHYTOME meat]). A) In combination with low drinking water nitrate levels. *p < 0.05; ***p < 0.001: ATNC levels after processed red meat intake significantly higher as compared to intake of white meat (*p* < 0.05), after intake of PHYTOME meat with standard levels of nitrite (*p* < 0.05), and after intake of PHYTOME meat with reduced levels of nitrite (*p* < 0.001). ATNC levels after white meat intake significantly higher as compared to levels after intake of PHYTOME meat with reduced‐nitrite levels (*p* < 0.05); B) In combination with drinking water nitrate levels at the acceptable daily intake level of 3.7 mg kg^−1^ body weight. *p < 0.05; ** *p* < 0.01: ATNC levels after intake of processed red meat combined with drinking water containing high levels of nitrate significantly higher as compared to ATNC levels after white meat consumption combined with drinking water containing low nitrate levels (*p* < 0.05), and significantly higher as compared to intake of PHYTOME meat with reduced‐nitrite levels combined with drinking water containing high nitrate levels (*p* < 0.05).

ATNC levels were measured in faecal waters and data for the complete intervention study (all time points) were available for 52 subjects (**Table** **3**). ATNC levels were significantly different between the groups (repeated measures ANOVA, *p* < 0.05). Using paired *t*‐tests, no statistically significant differences were found between baseline ATNC and ATNC after the different meat interventions. However, ATNC levels were significantly higher following processed red meat intake when compared to white meat intake (*p* < 0.05), standard‐nitrite PHYTOME meat intake (*p* < 0.05), and intake of PHYTOME meat with reduced levels of nitrite (*p* < 0.001). Furthermore, intake of white meat resulted in significantly higher levels of ATNC as compared to consumption of PHYTOME meat with reduced levels of nitrite (*p* < 0.05) (**Figure** **2**A).

**Table 3:** Fecal water apparent total N‐nitroso compounds (ATNC) levels, DNA strand breaks, and DNA adduct levels (O^6^‐methylguanine [O^6^‐MeG]: only for subjects with standard levels of nitrate in drinking water) of subjects consuming different meat products for 2 weeks in combination with a normal or high (acceptable daily intake [ADI] level of 3.7 mg kg^−1^ body weight) nitrate levels in drinking water (mean [SD]); for DNA strand breaks: first line value represents mean (SD), second line value represents median (SD)

Drinking water nitrate levels	Marker (unit)	Baseline	Processed red meat group	White meat group	PHYTOME meat group standard‐nitrite	PHYTOME meat group reduced‐nitrite
Normal (low)	ATNC [µmol L^−1^]^a)^	11.5 (18.8)	16.1 (8.6)^b)^	12.3 (11.5)^c)^	10.8 (11.8)	7.4 (4.8)
	DNA strand breaks (tail moment)^d)^	1.25 (0.91) 0.52 (0.57)	1.07 (0.47) 0.40 (0.23)	0.92 (0.44)^e)^ 0.34 (0.22)	0.99 (0.40) 0.33 (0.14)	1.02 (0.75) 0.54 (0.62)
	O^6^‐MeG adducts [pg per µg DNA]^f)^	2.0 (1.0)^g)^	2.6 (0.9)	1.4 (0.7)^h)^	2.4 (0.4)	2.6 (0.9)
High (ADI level)	ATNC [µmol L^−1^]	N.A.	21.0 (12.8)^i)^	17.6 (12.1)	17.8 (24.1)	10.3 (7.9)
	DNA strand breaks (tail moment)	N.A.	1.06 (0.41) 0.42 (0.26)	1.05 (0.24) 0.40 (0.17)	1.07 (0.63) 0.40 (0.33)	0.86 (0.33) 0.33 (0.15)

^a)^ Significantly different between the groups (repeated measures ANOVA, *p* < 0.05);

^b)^ Significantly higher as compared to white meat group (pairwise *t*‐test, *p* < 0.05), PHYTOME meat group standard‐nitrite (pairwise *t*‐test, *p* < 0.05), and PHYTOME meat group reduced‐nitrite (pairwise *t*‐test, *p* < 0.001);

^c)^ Significantly higher as compared to PHYTOME meat group reduced‐nitrite (pairwise *t*‐test, *p* < 0.05);

^d)^ Significantly different between the groups (repeated measures ANOVA, *p* < 0.05 [for mean TM]);

^e)^ Significantly lower as compared to baseline (pairwise *t*‐test, *p* < 0.01), and after 2 weeks of processed red meat consumption (pairwise *t*‐test, *p* < 0.01);

^f)^ Significantly different between the groups (repeated measures ANOVA, *p* < 0.001);

^g)^ Significantly lower as compared to processed red meat group (pairwise *t*‐test, *p* < 0.01), and as compared to PHYTOME meat group reduced‐nitrite (pairwise *t*‐test, *p* < 0.001);

^h)^ Significantly lower as compared to baseline (pairwise *t*‐test, *p* < 0.01), as compared to processed red meat group (pairwise t‐test, *p* < 0.001), as compared to PHYTOME meat standard‐nitrite group (pairwise *t*‐test, *p* < 0.001), and as compared to PHYTOME meat group reduced‐nitrite (pairwise *t*‐test, *p* < 0.001);

^i)^ Significantly higher as compared to white meat group combined with drinking water containing low nitrate levels (Student's *t*‐test, *p* < 0.05), and PHYTOME meat group reduced‐nitrite combined with drinking water containing high nitrate levels (Student's *t*‐test, *p* < 0.05).

ATNC levels after combined intake of the different meat types and drinking water nitrate levels at the ADI levels are displayed in Figure 2B. The consumption of processed red meat in combination with drinking water with high nitrate levels resulted in higher ATNC levels as compared to consumption of white meat (*p* < 0.05) combined with drinking water containing low nitrate levels and as compared to consumption of PHYTOME meat with reduced levels of nitrite combined with drinking water with high nitrate levels (*p* < 0.01). No significant differences were found between ATNC levels after white meat intake in combination with low nitrate levels in drinking water and after intake of PHYTOME meat combined with high levels of nitrate in drinking water.

In order to establish differences in ATNC excretion between high and low drinking water nitrate for each period of meat products separately, we have calculated the ATNC ratio for each participant for consumption of high and low levels of nitrate in drinking water. The ratio was significantly higher than 1 (single‐sided *t*‐test, *p* < 0.05) for all interventions except for the processed red meat. The mean ratios were 1.9 (95% CI: 1.1–2.6) for PHYTOME meat with standard‐nitrite levels, 2.6 (95% CI 1.1–4.2) for PHYTOME meat with reduced‐nitrite levels, and 2.3 (95% CI 1.3–3.3) for white meat. For processed red meat, the mean ratio was 1.9 (95% CI: 0.9–2.8), but this was not statistically significant. These results show that nitrate at the ADI level in drinking water stimulates the formation of NOCs.

### 3.3 Genotoxicity of Faecal Water

A significant difference was observed between the different intervention groups (repeated measures ANOVA, *p* < 0.05). The highest level of genotoxicity in Caco‐2 cells exposed to faecal water is found at the start of the study (baseline) (Table 3). At baseline and after 2 weeks of consumption of the processed red meat products (processed red meat group), the capacity to induce DNA strand breaks was significantly higher as compared to the faecal genotoxicity in the control period when only white meat was consumed (*p* < 0.01). No statistically significant differences were observed between the genotoxicity of faecal water after consumption of meat containing the added phytochemicals (standard‐nitrite PHYTOME meat and reduced‐nitrite PHYTOME meat) as compared to consumption of white meat. No additional effect of different nitrate levels in drinking water on genotoxicity was observed.

### 3.4 DNA Adduct Levels in Colonic Tissue

#### 3.4.1 O^6^‐MeG DNA Adduct Levels

The levels of O^6^‐MeG DNA adduct in colonic tissue were significantly different between the different interventions (repeated measures ANOVA, *p* < 0.001) (Table 3). At baseline, O^6^‐MeG DNA adduct levels in colonic tissue were significantly lower as compared to the levels after 2 weeks of consumption of the processed red meat products (*p* < 0.01), and as compared to PHYTOME meat products with reduced nitrite levels (*p* < 0.001). The O^6^‐MeG DNA adduct in colonic tissue were lowest after 2 weeks of white meat consumption (white vs baseline: *p* < 0.001; white vs processed red meat: *p* < 0.001; white vs PHYTOME meat standard‐nitrite: *p* < 0.001; white vs PHYTOME meat reduced‐nitrite *p* < 0.001).

### 3.5 Gene Expression Analyses

#### 3.5.1 Differentially Expressed Genes after Consumption of Different Meat Products

Differentially expressed genes (DEGs) after consumption of different meat types were identified by LIMMA analysis. DEGs were identified as compared to baseline levels for the processed red meat group, that is, 12 genes, counting 7 up‐ and 5 downregulated genes; for the PHYTOME meat group, that is, 170 DEGs, including 61 up‐ and 109 downregulated genes; and, for the standard‐nitrite PHTYTOME meat group, including only one downregulated gene. Detailed information on the DEGs is provided in Tables S2A–C, Supporting Information.

As the number of DEGs identified for the processed red meat group and the standard‐nitrite PHYTOME meat group are low, no pathway analyses were performed, but instead the function of all DEGs and their relation to CRC development and/or phytochemicals were explored using the NCBI portal (https://www.ncbi.nlm.nih.gov/gene) and a literature review, respectively (overview provided in Table S3A,B, Supporting Information). Four genes which were modulated in the processed red meat group, that is, gremlin 2, DAN family BMP antagonist (*GREM2*), carbonic anhydrase VII (*CA7*), aldo‐keto reductase family 1, member B10 (aldose reductase) (*AKR1B10*), and RAS protein activator like 1 (GAP1 like) (*RASAL1*) are reported to play a role in the colon and/or CRC and will be further described in the discussion.

**Figure 3:**
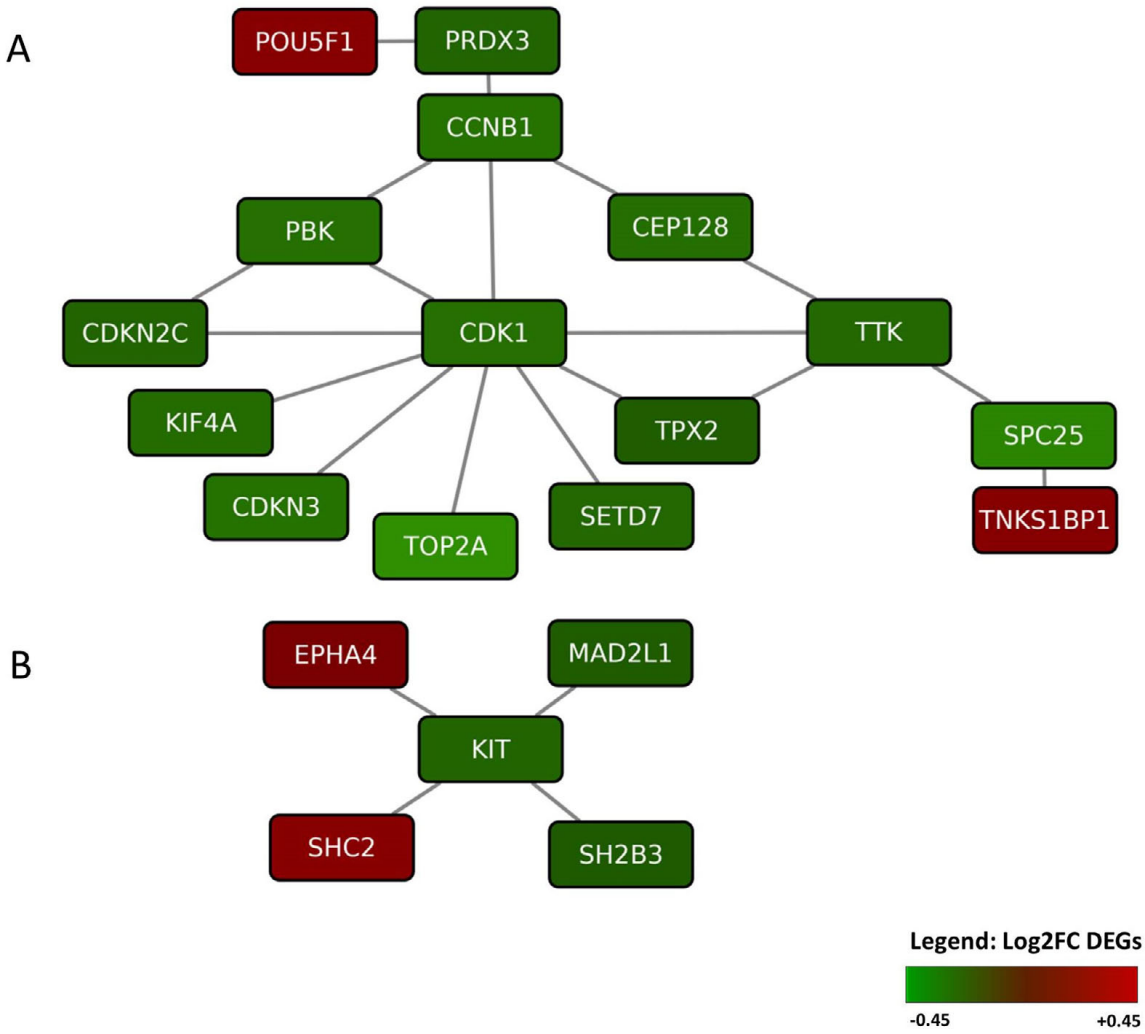
Network presentation of differentially expressed genes (DEGs) in colonic tissue of subjects (n = 57) consuming 2 weeks of processed red meat enriched with phytochemicals (PHYTOME meat) compared to baseline levels. A) Network of 15 unique DEGs containing 18 biological interactions; B) Network of five unique DEGs showing four biological interactions (Table S3D,E, Supporting Information). Networks were generated in Cytoscape^[^ ^59^ ^]^ based on the outcome of the induced network module application in ConsensupathDB (www.http://consensuspathdb.org/)^[^ ^58^ ^]^ using 170 unique DEGs as input list (Table S2B, Supporting Information). Fill color of the nodes represent control‐corrected log_2_ fold changes of DEGs Red: upregulation; Green: downregulation.

Overrepresentation analyses of the 170 DEGs found for the PHYTOME meat group as compared to baseline, identified 63 significantly modulated biological processes which are mostly related to cell cycle, the complement and coagulation system, and cholesterol biosynthesis (Table S3C, Supporting Information). Differentially expressed genes were used as input for creating a network which identified the known biological interactions between these DEGs. One large network consisting of 15 genes and one small network consisting of five genes was generated (**Figure** **3**A,B, Tables S3D,E, Supporting Information). Central in the large network, the cyclin‐dependent kinase 1 (*CDK1*) gene was connected to nine other genes, including cyclin B1 (*CCNB1)*, PDZ binding kinase (*PBK)*, cyclin‐dependent kinase inhibitor 2C (*CDKN2C*), kinesin family member 4A (*KIF4A*), cyclin‐dependent kinase inhibitor 3 (*CDKN3*), topoisomerase (DNA) II alpha (*TOP2A*), SET domain containing (lysine methyltransferase) 7 (*SETD7*), TPX2, microtubule‐associated (*TPX2*), and TTK protein kinase (*TTK*) (all downregulated). The small network consisted of one central gene, that is, KIT proto‐oncogene receptor tyrosine kinase (*KIT*, downregulated), which was linked to the four other genes, that is, EPH receptor A4 (*EPHA4*) and SHC (Src homology 2 domain containing) transforming protein 2 (*SHC2*), (upregulated), and MAD2 mitotic arrest deficient‐like 1 (*MAD2L1*) and SH2B adaptor protein 3 (*SH2B3*) (downregulated).

#### 3.5.2 Genes Associated with Different Exposure Conditions

Linear mixed model analyses were carried out in order to generate lists of significant genes associated with ATNC excretion, with the presence of phytochemicals, and with the interaction between the two. At an FDR < 0.05, one model gave significant results, showing 25 genes which were significantly associated with ATNC exposure (Table S2D, Supporting Information), including 13 negatively and 12 positively correlating genes. Two significantly modulated pathways involving serine and glycine biosynthesis, each containing two overrepresented genes, that is, vacuolar protein sorting 29 homolog (*Saccharomyces cerevisiae*) (*VPS29*) and phosphoserine phosphatase (*PSPH*) (overview provided in Table S3F, Supporting Information) were identified by pathway analyses. By using a less strict FDR cut off (FDR < 0.15), 249 significant genes were identified associated with the addition of phytochemicals to the diet, of which 144 were positively correlated and 105 were negatively correlated (Table S2E, Supporting Information). Genes are overrepresented in 121 pathways, involving mainly cell cycle and DNA repair related biological processes (ConsensuspathDB, FDR < 0.01) (Table S3G, Supporting Information). A large network could be generated comprising 86 genes demonstrating 186 connections. In addition, two smaller networks of seven and five genes respectively, were created (Table S3H,I, Supporting Information, **Figure** **4**A). Seven genes, all negatively correlated, in the large network connected to more than 10 other genes, that is, cyclin‐dependent kinase 2 (*CDK2*) (38 connections), *CDK1* (36 connections), replication protein A1 (*RPA1*), BRCA1 associated RING domain 1 *(BARD1)* (both 15 connections), protein phosphatase 1, catalytic subunit, gamma isozyme (*PPP1CC*) (12 connections), retinoblastoma 1 (*RB1*), and proliferating cell nuclear antigen (*PCNA*) (both 11 connections). Creation of a subnetwork of these seven genes with their interacting genes demonstrated that these seven genes highly interacted with each other directly, or via an intermediate gene (Table S3J,K, Supporting Information, Figure 4B). In addition to the identification of genes significantly associated with the addition of phytochemicals in the diet, the linear mixed model analyzing the interaction of ATNC and yes/no phytochemicals identified 214 significantly associated genes, of which 49 were negatively and 165 were positively correlated (Table S2F, Supporting Information). These genes are mainly involved in the biological processes glycolysis and gluconeogenesis, as shown by pathway analyses (ConsensuspathDB, FDR < 0.01) (Table S3L, Supporting Information).

Figure 4Network presentation of significantly associated genes in colonic tissue of subjects (n = 52) with the presence of phytochemicals in the diet. A) Large network of 86 genes containing 184 interactions, and two small networks of seven and five genes, respectively, showing six and four interactions (Table S3G–I, Supporting Information); B) Subnetwork of 50 genes containing 104 interactions (Table S3J,K, Supporting Information) extracted from the large network in (A). The seven genes presented in dashed black circles were connected to more than 10 other genes in the large network, that is, cyclin‐dependent kinase 2 (CDK2) (38 connections), CDK1 (36 connections), replication protein A1 (RPA1), BRCA1 associated RING domain 1 (BARD1) (both 15 connections), protein phosphatase 1, catalytic subunit, gamma isozyme (PPP1CC) (12 connections), retinoblastoma 1 (RB1), and proliferating cell nuclear antigen (PCNA) (both 11 connections). Networks were generated in Cytoscape^[^ ^59^ ^]^ based on the outcome of the induced network module application in ConsensupathDB (http://www.consensuspathdb.org/)^[^ ^58^ ^]^ using the 244 significant genes in colonic tissue of subjects associated with the presence of phytochemicals in the diet. Fill color of the nodes represent log_2_ regression coefficients. Red: positive regression coefficient; Green: negative regression coefficient.

**Figure:**
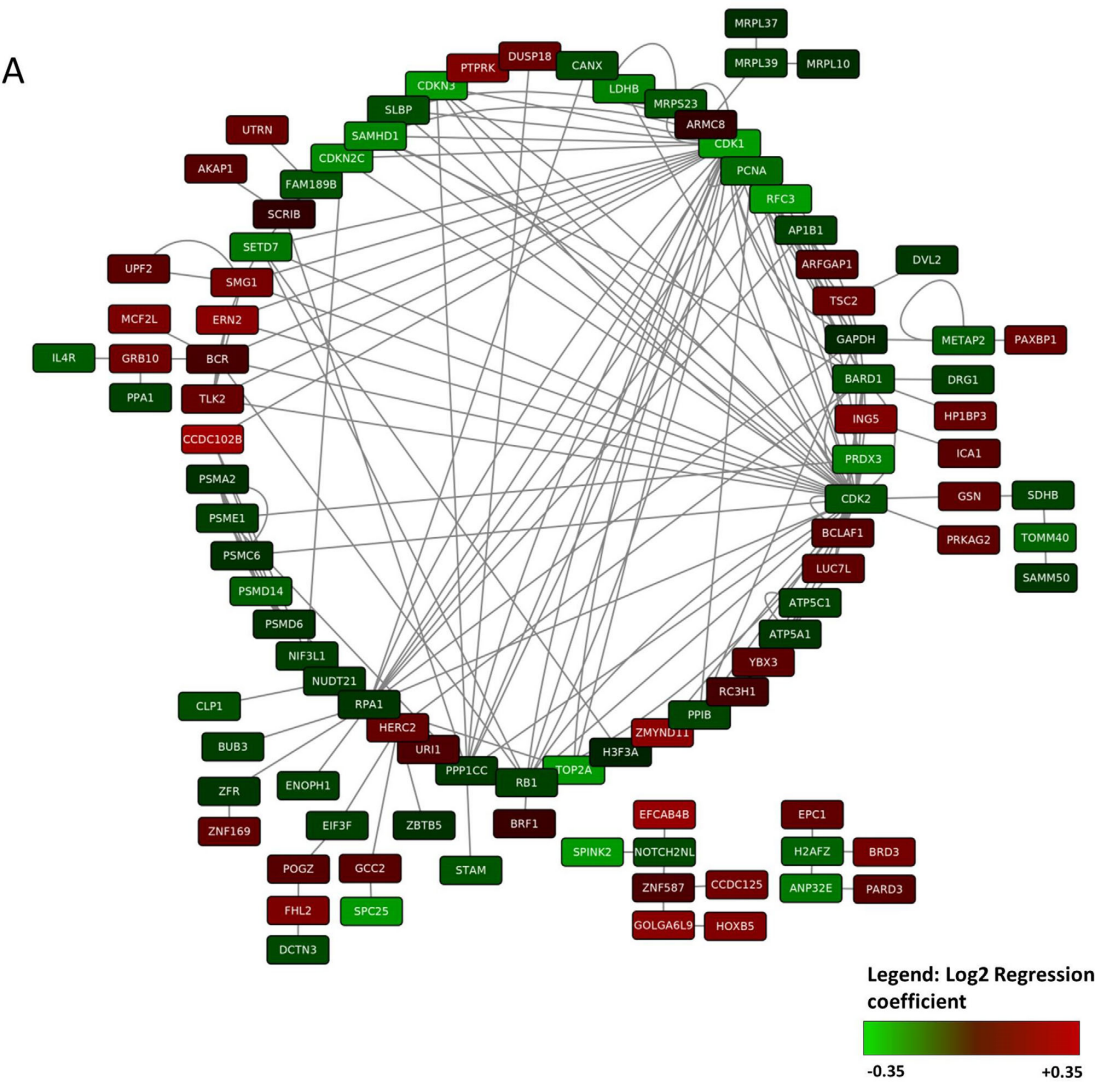

**Figure:**
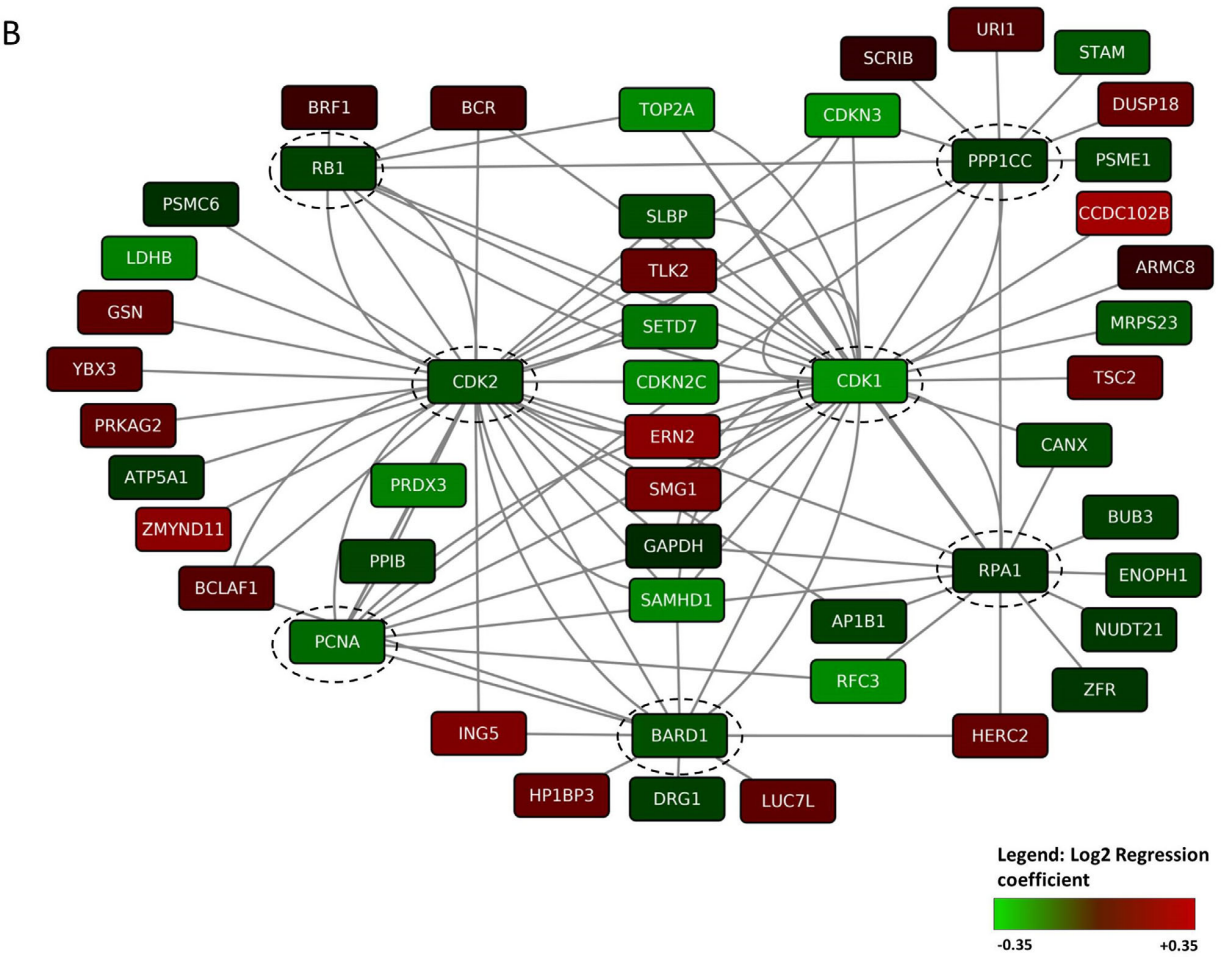

## 4 Discussion

In the PHYTOME project, we aimed to evaluate the impact of reformulated processed meat products enriched with natural bioactive compounds on the exposure levels of potentially carcinogenic NOCs in humans. The main finding of this human dietary intervention study is that consumption of PHYTOME meat products leads to a significant reduction in fecal ATNC levels, a surrogate marker of endogenously formed NOCs,^[^ ^9^ ^]^ as compared to the consumption of conventional processed red meat products. This outcome shows that the addition of natural plant extracts during red meat processing can reduce the endogenous formation of NOCs, even without the simultaneous reduction of nitrite added as a preservative, as the only difference between the conventional meat products and the PHYTOME meat products with standard nitrite levels are the added phytochemicals. The observed ATNC concentrations in faecal water are comparable to those found after consumption of white meat. Addition of natural extracts in combination with reduction of nitrite lowered the level even further, as compared to the level of ATNC after consumption of white meat. There is no additional signficant reduction in ATNC levels in faecal water after consumption of PHYTOME meat with reduced levels of nitrite as compared to the level after consumption of PHYTOME meat with standard levels of nitrite. Notably, the level of significance is higher in the comparison between processed red meat and processed red meat with reduced levels of nitrite and added phytochemicals as compared to the comparison between processed red meat and processed red meat with standard levels of nitrite and added phytochemicals. This indicates that reduction of nitrite increases the strength of evidence in probabilistic terms. As the exposure to NOCs is one of the suggested mechanisms by which red and processed red meat can increase the risk of CRC,^[^ ^3^ ^]^ a reduction in their formation is likely to have a beneficial effect.

On the other hand, increasing drinking water nitrate to the level of the ADI is found to stimulate the formation of ATNC, specifically in combination with conventionally processed red meat products. ATNC levels also tended to be higher after the combined intake of drinking water containing nitrate at the ADI level and white meat. These findings confirm the results of our pilot study in which we investigated the contribution of nitrate in drinking water in combination with either consumption of processed red meat or white meat on endogenous NOC formation.^[^ ^36^ ^]^ The combination of reduced‐nitrite in the PHYTOME meat and low levels of nitrate in drinking water induced the largest reduction on the endogenous formation of NOCs. The results from the current study indicates that consumption of nitrite and nitrate enhances endogenous nitrosation, but that addition of phytochemicals can significantly inhibit this. These findings imply that dietary intake of both nitrite and nitrate from other sources should be taken into consideration when cancer risk assessments are made for the consumption of meat products.

By fecal water genotoxicity measurements, it was confirmed that the consumption of processed red meat products increased DNA strand breaks in colonic cells as compared to the consumption of white meat. These results are in line with those from a previously conducted human dietary intervention study in which patients with either inflammatory bowel disease or irritable bowel syndrome consumed a high red meat diet consisting of 300 g of red meat per day for 7 days.^[^ ^19^ ^]^ After the intervention, mean levels of DNA strand breaks induced ex vivo in Caco‐2 cells by fecal water were significantly higher. In the present study, no statistically significant difference in the level of DNA strand breaks was found after PHYTOME meat consumption as compared to consumption of white meat or processed red meat, either with or without nitrite reduction, indicating no difference in genotoxic potential between PHYTOME meat products and conventional processed red meat products or white meat.

O^6^‐methylguanine adducts levels in colonic tissue were lowest after consumption of white meat. The adduct levels were significantly higher at baseline, after consumption of conventionally processed red meat products, and after consumption of the PHYTOME meat products, as compared to white meat. Previous studies have shown that O^6^‐methylguanine adducts are present in CRC tissue, and several in vitro^[^ ^60^ ^]^ and animal in vivo^[^ ^61^, ^62^ ^]^ studies have demonstrated an increase in the number of adducts after NOC exposure. This is the first human intervention study that reports the increase of this adduct in colon tissue after an intervention with processed red meat. Although exposure to ATNC was reduced in the subjects after consumption of the PHYTOME meat products, this did not result in a lower O^6^‐methylguanine adduct level in their colon.

Gene expression analyses were carried out in colonic tissue in order to elucidate the molecular mechanisms that are affected by the dietary intervention. It was shown that consumption of processed red meat modulated the expression 12 genes in colonic tissue of which three are known to be involved in CRC development, that is, *CA7, AKR1B10*, and *RASAL1*, and of which one plays a role in normal colonic tissue differentiation, that is, *GREM2*. The downregulated *CA7* gene encodes a metallo‐enzyme which catalyses the conversion of CO_2_ to the bicarbonate ion and protons. This reaction is involved in many physiological and pathological reactions, including tumourigenicity.^[^ ^63^ ^]^ Clinical studies have shown that CA7 expression is downregulated in CRC tissue both at the mRNA and protein level. Furthermore, decreased expression of CA7 correlates with CRC progression and a poor clinical prognosis.^[^ ^64^ ^]^ Suggested mechanisms involve dedifferentiation^[^ ^64^ ^]^ and reduced protection against oxidative stress.^[^ ^65^ ^]^ *AKR1B10*, which was also downregulated after processed red meat consumption in the current study, is specifically expressed in the small intestine and colon, and identified as a direct target of p53.^[^ ^66^ ^]^ It is member of the aldo‐keto reductase superfamily and encodes for an enzyme that catalyses the reduction of electrophilic carbonyl compounds to less toxic alcoholic metabolites, thereby protecting the intestinal cell against DNA damage.^[^ ^67^ ^]^ Several studies have reported a decreased expression level of *AKR1B10* in colorectal carcinomas.^[^ ^66^, ^68^, ^69^ ^]^ In particular, it was lost or decreased in CRC tissue, precancerous tissue, and even in normal adjacent tissue.^[^ ^69^ ^]^ Possible mechanisms by which AKR1B10 might contribute to CRC development and poor clinical prognosis comprise inhibition of p53‐induced apoptosis and loss of proliferative suppression of CRC cells.^[^ ^66^ ^]^ *RASAL1* was upregulated in the colon of subjects consuming processed red meat. The protein product of this gene controls cellular proliferation and differentiation by suppressing the normal RAS function. It was found to be overexpressed in CRC cells with the mutant KRAS variant. Furthermore, it was suggested that RASAL1 functions in the progression of benign colonic neoplasms.^[^ ^70^ ^]^ Therefore, in subjects who are at risk for CRC and bear benign polyps in their intestine containing mutated KRAS genes, upregulation of this gene could lead to progression to advanced malignant lesions. In addition to these three genes of which is reported that their expression is changed in CRC tissue, the expression of *GREM2* was downregulated. The protein product of this gene is an antagonist of BMP (bone morphogenic protein) signaling, and expression of *GREM2* contributes to inhibition of differentiation of basal crypt epithelial cells.^[^ ^71^ ^]^ A role of BMP signaling has been suggested in the inhibition of self‐renewal of intestinal stem cells by inhibition of Wnt signaling.^[^ ^72^ ^]^ Therefore, aberrations in the expression of genes involved in BMP signaling which plays an important role in the correct development of the colonic crypt are not desired. In conclusion, subjects consuming processed red meat show colonic gene expressions that are linked to increased cell proliferation and dedifferentiation.

Subjects consuming processed red meat excreted highest ATNC levels and linear mixed model analyses identified two genes associated with ATNC exposure, that is, *PSPH* (positively correlated), and *VPS29* (negatively correlated), both overrepresented in the serine and glycine biosynthesis pathway. Serine and glycine (which can be formed out of serine) are important amino acids with a role in cancer cell metabolism by acting as precursors for synthesis of proteins, nucleic acids, and lipids which are important for cancer cell growth.^[^ ^73^ ^]^ With increasing ATNC exposure, the expression of *PSPH* also increased, potentially stimulating serine formation, thereby contributing to synthesis of building blocks for increasing cell growth. In a previous human dietary intervention study performed by our group, in which human healthy volunteers consumed 300 g red meat per day for 7 days, genes significantly correlating with the increase in fecal water genotoxicity were involved in similar biological pathways as found in the current study, including cell cycle and WNT signaling.^[^ ^19^ ^]^ This emphasizes the possible contribution of these processes in red meat induced CRC risk.

Cell proliferation is also the main affected biological process on gene expression level in colonic tissue of subjects consuming the PHYTOME meat. *CDK1* (downregulated), the central gene in a large network of differentially expressed genes, is a key player in cell cycle regulation (Figure 3A). It forms a complex with CCNB1 (downregulated), which promotes several events in mitosis. It is responsible for phosphorylation of PBK (downregulated) during mitosis which is required for its mitotic activity.^[^ ^74^ ^]^ In addition to its role in cytokinesis during mitosis, overexpressed PBK is found in numerous cancers such as colorectal cancer,^[^ ^75^ ^]^ bladder cancer,^[^ ^76^ ^]^ and gastric cancer.^[^ ^77^ ^]^ In addition to *CCNB1*, *CDK1* is linked to other genes involved in cell cycle regulation in the network, such as *TOP2A*, SPC25, NDC80 kinetochore complex component (*SPC25*), *CDKN3*, *KIF4A*, *TTK*, *CDKN2C*, and *TPX2* (all downregulated), and tankyrase 1 binding protein 1 (*TNKS1BP1*) (upregulated). Based on the function of the protein products of these genes, these modulations will have an inhibitory effect on cell proliferation. Furthermore, overexpression of *CDK1*,^[^ ^78^ ^]^ *CCNB1*,^[^ ^79^ ^]^ peroxiredoxin 3 (*PRDX3)*,^[^ ^80^ ^]^ *TOP2A*,^[^ ^81^ ^]^ *SPC25*,^[^ ^82^ ^]^ *CDKN3*,^[^ ^83^ ^]^ *KIF4A*,^[^ ^84^ ^]^ *TTK*,^[^ ^85^ ^]^ POU class 5 homeobox 1 (*POU5F1*),^[^ ^86^ ^]^ and *TPX2* ^[^ ^87^ ^]^ has been reported to play a role in CRC. As the expression of these genes was downregulated, except for *POU5F1*, these modulations are in favor of protection against CRC. Overall, these results suggest that consumption of meat containing phytochemicals may inhibit the process of cell proliferation.

In line with these findings, linear mixed model analyses identified genes associated with exposure to phytochemicals to play in particular a role in the regulation of the cell cycle. This was predominantly mediated by *CDK1*, *CDK2*, *BARD1*, *RPA1*, *RB1*, *PCNA*, and *PPP1CC* (Figure 4A,B). CDK1 was also identified as central gene in the network of genes differentially expressed in the colon of subjects consuming PHYTOME meat enriched with phytochemicals, as discussed before. In addition to CDK1, also TOP2A, CDKN2C, SETD7, CDKN3, and PRDX3, were present in both networks (Figures 3A and 4), which demonstrates the relevance of these gene expression changes. Both CDK1 and CDK2 are responsible for phosphorylation of RB1, leading to its inactivation and subsequent progression of the cell cycle.^[^ ^88^ ^]^ In contrast, PPP1CC, which is a positive regulator of cell growth, is responsible for dephosphorylation of RB1, in particular after transition of the cell cycle to the M‐G1 stage.^[^ ^89^ ^]^ Although RB1 is regarded as a tumour suppressor gene, and loss of RB1 function and either low or no expression has been shown in different tumour types, RB1 expression was upregulated in colorectal carcinomas at both the mRNA and protein levels.^[^ ^90^, ^91^, ^92^ ^]^ It is suggested that RB1 plays a role in colorectal tumorigenesis through functional regulation of the transcript and protein rather than through its tumour suppressor role by gene inactivation.^[^ ^92^ ^]^ In addition to phosphorylation of RB1, CDK1 and CDK2 phosphorylate RPA1.^[^ ^93^ ^]^ RPA1 is essential for DNA replication, repair, and recombination. However, in numerous cancers including CRC,^[^ ^94^, ^95^, ^96^, ^97^, ^98^ ^]^ RPA1 mRNA and/or protein expression is significantly increased and associated with a poor prognosis in advanced cancer patients and known to stimulate proliferation of cancer cells^[^ ^97^ ^]^ . RPA1 is also involved in mono ubiquitination of PCNA. In addition to its function in DNA repair, PCNA forms complexes with all CDK‐cyclin complexes during the cell cycle, inducing both positive (among which CDK1‐cyclin A and CDK2‐cyclin E) and negative effects on progression.^[^ ^99^ ^]^ High PCNA expression was associated with poor clinical outcome of CRC, and could be used as a biomarker for clinical prognosis.^[^ ^100^ ^]^ Additionally, PCNA interacts with BARD1 and in this way participates in the cellular response to DNA damage.^[^ ^101^ ^]^ Both CDK2‐cyclin A1/E1 and CDK1‐cyclin B1 have been shown to phosphorylate BARD1, leading to its accumulation in the M‐phase.^[^ ^102^ ^]^ In CRC patients, loss of full length protein BARD1 was associated with poor survival rates, and demonstrates its tumor suppressive function.^[^ ^103^ ^]^ Overall, these effects on the cell cycle related genes *CDK1, CDK2, RPA1, RB1, PCNA*, and *PPP1CC*, except for the effect on *BARD1* expression, are likely to result in reduced colonic cell proliferation. In addition, they play a role in different phases of the cell cycle, indicating that addition of phytochemicals via meat products impacts cell proliferation at multiple levels. Our results are in line with those reported by Bailon‐Moscoso et al.^[^ ^104^ ^]^ who show that cell cycle related genes, in particular CDKs and cyclins, are a key target of phytochemicals.

In summary, the whole genome gene expression analyses identified differentially expressed genes and genes associated with ATNC levels with or without simultaneous exposure to phytochemicals, which are related to molecular pathways which may explain cancer risk initiation after intake of processed red meat and cancer risk prevention after intake of the PHYTOME meat. In particular, key genes that are significantly modulated in the colon of subjects consuming processed red meat and genes that are associated with ATNC levels are linked to processes stimulating cell proliferation and dedifferentiation, which mechanistically support cancer risk. In contrast, intake of PHYTOME meat products demonstrated a strong response on cell cycle related genes, which may lead to inhibition of cell proliferation, which is likely to have a preventive effect against CRC.

This study has a number of strong points, such as the parallel design including a control period in order to washout the effects induced by the processed red meat intervention before subjects receive the PHYTOME meat. Furthermore, diets were semi‐controlled by standardizing the amount of fruits and vegetables intake and the concentration of nitrate in drinking water throughout the whole study. The length of the intervention periods was sufficient and sample size was large enough in order to expect biologically relevant effects of different meat interventions including a reduction in nitrite levels on the measured markers. A limitation of the study was the lack of a washout period at the beginning of the study, which could have resulted in high inter‐individual differences at baseline. Furthermore, gene expression changes and effects on O^6^‐methyl‐guanine adduct levels were determined in colonic biopsies consisting of a mixture of different cell populations. The contribution of each of these different cell types on the measured effects is therefore not clear.

The outcome of this study demonstrates that exposure to meat‐induced NOC exposure is modifiable, but in order to have a positive health impact also a successful implementation is required. Consumers expressed a generally favorable attitude towards and relatively strong interest in the PHYTOME meat products,^[^ ^105^ ^]^ but they also flagged concerns about the actual healthiness and raised doubts whether the innovative processed meat products would actually be more than a marketing gimmick.^[^ ^23^ ^]^ These studies also demonstrated that initial responses of stakeholders and consumers towards the PHYTOME concept are generally favorable owing to the potential for improving processed meat products’ health image and perceived naturalness, respectively.^[^ ^23^ ^]^ As this study provides the scientific evidence for substantiation of the eventual health benefits of consuming these innovative meat products, the potential health impact of introducing the PHYTOME meat products to the market is promising.

Overall, the data from the human dietary intervention study support the rationale behind the PHYTOME concept, that is, that the introduction of natural bioactive compounds from plant extracts in combination with standard or reduced amounts of added nitrite in processed meat products, results in the reduced formation of NOCs and less adverse reactions in the human large intestine. Molecular mechanisms have been identified which may mechanistically support this finding. Furthermore, high drinking water nitrate was found to stimulate endogenous nitrosation. It is therefore crucial to take dietary intake of phytochemicals from fruits and vegetables, as well as drinking water nitrate into consideration when evaluating the risk of colon cancer risk associated with meat consumption. We propose that these newly developed meat products enriched with phytochemicals may be promising alternatives for conventional meat products, by improving gut health and reducing CRC risk as a result of a decreased exposure to intraluminal N‐nitroso compounds.

## Conflict of Interest

The authors declare no conflict of interest.

## Author Contributions

The authors’ contributions were as follows: S.B. has written the original draft of the paper. Reviewing and editing of the draft was done by T.K., G.K., R.S., S.B., G.H., J.H., V.W., and G.S. T.K. was responsible for conceptualization of the whole study in collaboration with G.K., R.S. Writing of the METC protocol was done by K.M. Conduction of the trial, enrollment of subjects, assignment of participant to interventions, and specimen collection was done by K.M. and she was assisted by H.P. Sampling of biopsies by means of endoscopy was done by A.B., assisted by C.V., and by A.M. Supervision of the trial was carried out by T.K. Development of meat products was done by G.S., G.P., R.V. and the industrial consortium partners. Analysis of ATNC was done by V.S. and G.K. Analyses of DNA adducts was performed by P.G. Ex‐vivo genotoxicity was measured by H.P. DNA extraction was done by H.P. Data analyses and statistical analyses was done by S.B. in close collaboration with T.M., G.K., and R.S. Data interpretation was done by S.B., T.K., G.K., P.G., and R.S. All authors contributed to and approved the final manuscript.

## Supporting information


**Supplementary Figure 1** Flow of study participants.

**Supplementary Figure 2** Hierarchical clustering of microarray data for each subject at each dietary intervention. From 59 individuals, 4 were removed from the processed red meat group, and 2 were removed from the white meat group and PHYTOME meat group.

Supporting Information.

Supporting Information.

Supporting Information.

## Data Availability

Data described in the manuscript, code book, and analytic code will be made available upon request.
