# Supplementary figures and images for: Replacement of Nitrite in Meat Products by Natural Bioactive Compounds Results in Reduced Exposure to N‐Nitroso Compounds: The PHYTOME Project
Source: Mol Nutr Food Res. 2021 Aug 27;65(20):2001214. doi: 10.1002/mnfr.202001214 (PMC8530897; doi:10.1002/mnfr.202001214)

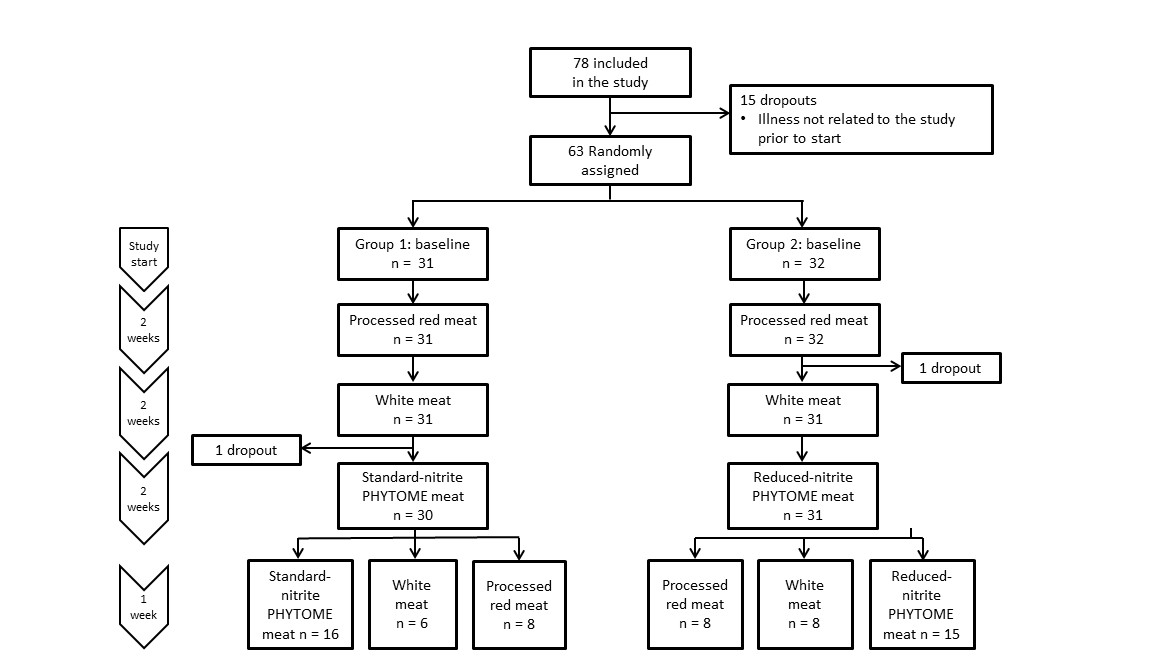

Supplement: Supplementary file 1 — Supplementary Figure 1 Flow of study participants. [file MNFR-65-0-s001.jpg]

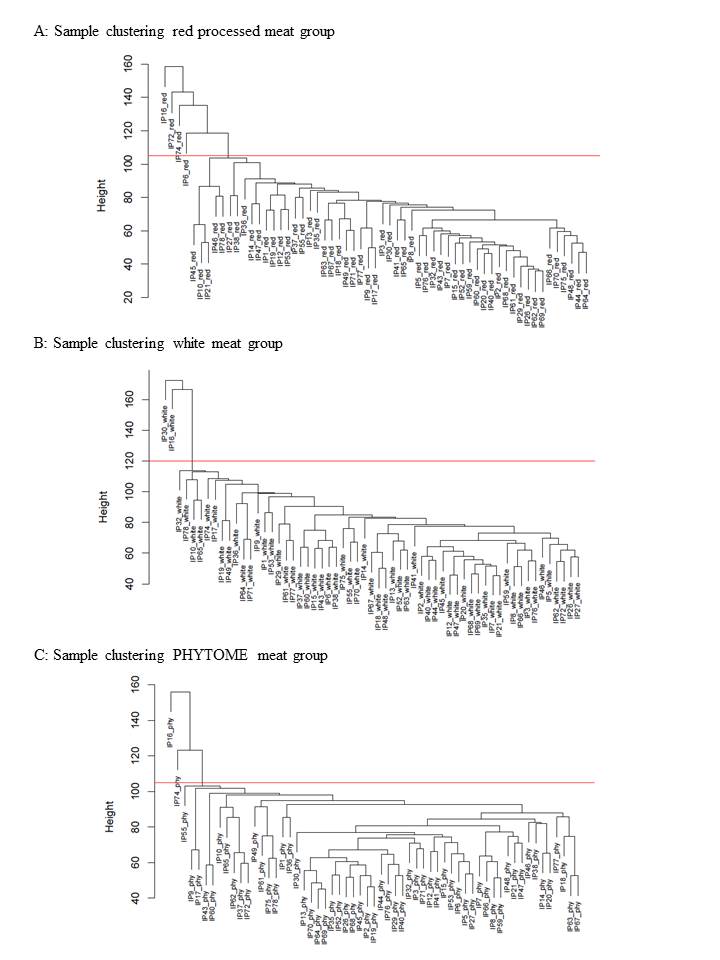

Supplement: Supplementary file 2 — Supplementary Figure 2 Hierarchical clustering of microarray data for each subject at each dietary intervention. From 59 individuals, 4 were removed from the processed red meat group, and 2 were removed from the white meat group and PHYTOME meat group. [file MNFR-65-0-s002.jpg]
