# Supplementary material for: Replacement of Nitrite in Meat Products by Natural Bioactive Compounds Results in Reduced Exposure to N‐Nitroso Compounds: The PHYTOME Project
Source: Mol Nutr Food Res. 2021 Aug 27;65(20):2001214. doi: 10.1002/mnfr.202001214 (PMC8530897; doi:10.1002/mnfr.202001214)
Supplement: Supplementary file 3 — Supporting Information. [file MNFR-65-0-s003.docx]

**Supplementary data Table 1** Mean (SD) daily dietary intake of energy and macronutrients in the different intervention periods

| **Daily dietary intake**  **Mean (SEM)** | **Period A**  **(Red processed meat)** | **Period O**  **(White meat)** | **Period B**  **(PHYTOME meat)** | **Period C**  **(High drinking water nitrate)** |
| --- | --- | --- | --- | --- |
| Energy (kcal)^a^ | 1902 (324) | 1935 (356) | 1979 (342) | 1879 (482) |
| Fat (g)^b^ | 84 (18) | 67 (18) ^f^ | 89 (19) | 80 (24) |
| Saturated fat (g)^c^ | 33 (9) | 26 (9) ^g^ | 34 (9) | 31 (10) |
| Carbohydrates (g)^d^ | 187 (38) | 209 (53) ^h^ | 194 (47) | 188 (64) |
| Protein (g)^e^ | 87 (21) | 115 (26) ^i^ | 90 (22) | 91 (28) |

^a^ not statistically significantly different; repeated measures ANOVA; n =27, F = 1.10, df = 3, p = 0.35

^b^ statistically significantly different; repeated measures ANOVA; n =27, F = 14.77, df = 3, p <0.001

^c^ statistically significantly different; repeated measures ANOVA; n =27, F = 10.88, df = 3, p < 0.001

^d^ statistically significantly different; repeated measures ANOVA; n =27, F = 3.00, df = 3, p = 0.036

^e^ statistically significantly different; repeated measures ANOVA; n =27, F = 36.38, df = 3, p < 0.001

^f^ statistically significantly lower as compared to period A (paired t-test, t = -6.66, df = 27, p < 0.001), period B (paired t-test, t = -8.29, df = 26, p < 0.001), and period C (paired t-test, t = -3.56, df = 27, p = 0.001)

^g^ statistically significantly lower as compared to period A (paired t-test, t = -5.02, df = 27, p < 0.001), period B (paired t-test, t = -7.83, df = 26, p < 0.001), and period C (paired t-test, t = -3.45, df = 27, p = 0.002)

^h^ statistically significantly higher as compared to period A (paired t-test, t = 3.12, df = 27, p = 0.004), and period C (paired t-test, t = 2.71, df = 27, p = 0.012)

^i^ statistically significantly higher as compared to period A (paired t-test, t = 6.18, df = 27, p < 0.001), period B (paired t-test, t = 8.30, df = 26, p < 0.001), and period C (paired t-test, t = 5.60, df = 27, p <0.001)
